# Defining (trained) grapheme-color synesthesia

**DOI:** 10.3389/fnhum.2014.00368

**Published:** 2014-06-04

**Authors:** Olympia Colizoli, Jaap M. J. Murre, Romke Rouw

**Affiliations:** Department of Psychology, Brain and Cognition, University of AmsterdamAmsterdam, Netherlands

**Keywords:** synesthesia, grapheme-color, training, development, learning, memory

There is a current debate over the possibility and validity of synesthesia training experiments (Deroy and Spence, 2013; Rothen and Meier, 2014). In order to test whether a trainee should be considered to have acquired a trained form of synesthesia, a precise definition and specific diagnostic criteria of *synesthesia* are necessary. There is currently not one specific checklist available including all specific diagnostic methods and criteria, exacerbating the determination and interpretation of differences between (potentially) trained and genuine synesthesia. In order to facilitate communication surrounding these issues, we propose a practical guideline for diagnosing the specific characteristics that are typical of *grapheme-color synesthesia* (GCS). These guidelines can be applied to developmental cases of GCS, cases of trainees who may show synesthetic traits, and other types of acquired forms of GCS at the level of a *single individual*.

Researchers have tried to train synesthesia since at least 1934 (Kelly, 1934), and several studies have been done in recent years (for reviews see: Deroy and Spence, 2013; Rothen and Meier, 2014). Leaving trained, acquired, or induced types of synesthesia aside, it is important to acknowledge the extent of inter-individual differences between synesthetes (even within one sub-type), making a generally accepted definition difficult to reach. For example, an individual may report that letters automatically induced the conscious experience of color since childhood, however, these colors may not be *consistently* mapped to each letter. Such an individual would fail the “test of genuineness,” while he or she may still meet the other defining characteristics of synesthesia, such as the conscious experience of color in the absence of physical color. This is one example of why the definition of *developmental synesthesia* is a topic of active debate in the literature (Cohen Kadosh and Terhune, 2012; Eagleman, 2012; Simner, 2012). The need for a consensus on the specific defining characteristics of synesthesia becomes only more prominent when trying to compare trainees to synesthetes.

Characteristics of GCS at the individual vs. group level must be distinguished. Group-level effects related to synesthesia cannot (yet) be used as diagnostic tools. If two groups differ *relatively* from each other on a certain score or measurement, this does not tell us at an *absolute* level whether “score X on task A” or “measurement Y in trait B” of an individual implies that he or she is necessarily a synesthete. The nature of such studies does not allow one to assume that a certain measurement is *unique* to synesthesia. For example, it has been shown that GCS is associated with increased structural connectivity compared to controls in the temporal cortex at the *group-level* (Rouw and Scholte, 2007). However, the absolute value measured for white-matter connectivity in a particular individual could be influenced by different unknown causes. Although measurements can made at the individual level (see Table 1), experiments designed to test group-level characteristics related to GCS by definition cannot be used as diagnostic criteria, such as memory benefits (Yaro and Ward, 2007; Rothen and Meier, 2010; Rothen et al., 2012; Pritchard et al., 2013; Terhune et al., 2013), differences in visual processing (Terhune et al., 2011; Brang et al., 2012; Banissy et al., 2013), the reported vividness of visual mental imagery (Barnett and Newell, 2008), distinct cognitive styles (Meier and Rothen, 2013) or differences in neuroanatomy (for a review see: Rouw et al., 2011). Similarly, idiosyncrasy (the fact that grapheme-color mappings differ *between* individuals) cannot be used as a marker at the individual level; if two synesthetes have nearly identical mappings, they are not excluded from being considered synesthetes (Witthoft and Winawer, 2013). Additionally, the low prevalence of synesthesia is a group-level characteristic that is not diagnostic at the individual level. Most neurobiological markers related to GCS (Rouw et al., 2011) and the presence of certain genetic markers (Asher et al., 2009; Tomson et al., 2011) are related to the group-level. Individual-level traits are sometimes confused with inclusive criteria for GCS. For example, GCS *tends* to be unidirectional at a conscious level (i.e., graphemes elicit color experiences, but color experiences do not elicit conscious grapheme experiences) and bidirectional at an unconscious level (Knoch et al., 2005; Cohen Kadosh and Henik, 2006; Cohen Kadosh et al., 2007; Gebuis et al., 2009; Weiss et al., 2009; Rothen et al., 2010). Still, an instance of *conscious* bidirectional GCS does not exclude an individual from being considered a synesthete when the other defining characteristics are met (e.g., Cohen Kadosh et al., 2007).

**Table 1:** **Proposed diagnostic criteria for grapheme-color synesthesia at the level of a single individual**.

	**Dimension**	**Method**	**Criteria**	**Remarks**
Major axis grapheme-color synesthesia	Consistent	*Percentage within a duration:* Test–retest paradigm using the Cambridge Synesthesia Charts (TOG: (Baron-Cohen et al., 1987); TOG-R: Asher et al., 2006)	*Percentage within a duration:* 70–100% after at least 3 months (Baron-Cohen et al., 1987; Asher et al., 2006)	We suggest that especially for trained or acquired forms of synesthesia that the online color-picker consistency tests be repeated with more than 3 months in between testing sessions in order to exclude possible memory strategies that could be facilitated by training or temporary mental states such as can be induced under hypnosis. We note that there is open discussion on the criteria of consistency in the field (Cohen Kadosh and Terhune, 2012; Eagleman, 2012; Simner, 2012)
		*Color-distance level:* Online color pickers (Eagleman et al., 2007; Rothen et al., 2013a)	*Color-distance level:* Cut-off at 135 in CIELUV color space (Eagleman et al., 2007; Rothen et al., 2013a)	The Eagleman et al. (2007) test for consistency is completed within a single testing session, and the original criteria in RGB space (cut-off at 1) is also an acceptable level of consistency, although it has been shown to be too conservative (Rothen et al., 2013a). Euclidean distance in CIELUV color space was shown to have the best discriminatory power, because this space is perceptually uniform (Rothen et al., 2013a). Conversion software may be downloaded here: http://www.sussex.ac.uk/synesthesia/links
	Automatic	Synesthetic Stroop test	Using a within-subjects design, a significant difference in performance for incongruent compared to congruent stimuli should be found (Wollen and Ruggiero, 1983; Mills, 1999; Odgaard et al., 1999; Dixon et al., 2000; Smilek et al., 2001). For interpretation of synesthesia-related behavior, it is important to include a within-subject baseline measure, such as digit or letter naming in addition to (synesthetic) color naming (Mills, 1999), and especially when the case cannot be compared to a group of matched controls	Automaticity refers to the involuntary nature of the experience of the synesthetic concurrent, which elicits interference with task demands. It is important to note that the presence of a Stroop effect does not necessarily imply that the association is at a perceptual level
		Conditioned response	A conditioned response to the unconditioned grapheme stimulus should be found after the corresponding color of the grapheme, but should not be present in a control condition or group (Meier and Rothen, 2007; Rothen et al., 2010)	It has been argued that the synesthetic conditioned-response effect may alternatively reflect the conscious experience instead of the automatic nature of the synesthetic color, because it is only present in developmental synesthetes and not trainees who show reliable Stroop effects (Meier and Rothen, 2007, 2009; Rothen et al., 2010)
		Pupil diameter	The average pupil diameter measured when viewing incongruent graphemes is significantly larger compared to viewing congruent and black graphemes (Paulsen and Laeng, 2006)	Pupil diameter is a physiological measure of the autonomic nervous system (e.g., Weiskrantz, 1998). Reliable differences related to synesthetic congruency in pupil diameter were found at the individual and group levels (Paulsen and Laeng, 2006) It remains to be shown whether this difference in pupil diameter can be used as an index of conscious vs. unconscious processes related to grapheme-color synesthesia
		Validated questionnaire: CLaN (Rothen et al., 2013b)	A score >3 on questions pertaining to the automaticity of colors (note that some questions need to be reversed for the final scoring; Rothen et al., 2013b)	The degree of automaticity can vary between synesthetes and was shown to be correlated with levels of interference in a synesthetic-color naming Stroop task (Rothen et al., 2013b). The CLaN questionnaire can be downloaded here: http://www.sussex.ac.uk/synaesthesia/links
	Conscious	Interview/questionnaires	The individual should report conscious experiences of color at a perceptual level	An example questionnaire designed for trainees can be found in Colizoli et al. (2012); Colizoli et al. (2014). We follow Rothen and Meier (2014) that if an individual reports *knowing* that the letter has a color, but does not report a phenomenological experience of the color itself, this individual should not be considered to be a synesthete
	Perceptual	Crowding (Hubbard et al., 2005), Visual-Search (Palmeri et al., 2002), Opponent Color Effects (Nikolić et al., 2007), Binocularly Defined Stimuli (Palmeri et al., 2002; Kim et al., 2006), Motion-Defined Stimuli (Palmeri et al., 2002; Kim et al., 2006)	The individual should show significant low-level perceptual effects of color, in the absence of physical color. Evidence for the presence of perceptual effects of synesthesia in an individual is strongest when a group of matched controls does not show the effects tested	Due to the individual differences within synesthesia, it is not always the case that every “synesthete” will show significant low-level perceptual effects of color. For example, it was believed that grapheme-color synesthesia involved pre-attentive pop-out of color in the presence of inducing graphemes, however, this has been shown not to be the case for all synesthetes tested (e.g., Hubbard et al., 2005; Rothen and Meier, 2009; Ward et al., 2010; Rich and Karstoft, 2013)
		Case vs. control neuroimaging study (Elias et al., 2003; Steven et al., 2006)	The individual (case) but not the control group, should show differential neural activation or patterns of activation related to the concurrent sense when only the inducing sense is triggered. The control group should not show activation related to the concurrent sense when the inducing sense is triggered	We note that within grapheme-color synesthesia, dissociating the inducer (grapheme) and concurrent (color) modalities is challenging due to their physical proximity in the brain and the fact that both modalities are in the visual sense. For this reason, it is not always clear whether color activation is reliably found in the presence of inducing black graphemes (Rouw et al., 2011). Using auditory linguistic stimuli in contrast to visual stimuli may help to dissociate brain activation related to the inducer vs. the concurrent sense in grapheme-color synesthesia (given that the auditory mode elicits the visual synesthetic experience)
	Bandwidth	*Inducer-Bandwidth*: # Inducers/# Possible Inducers (Asher et al., 2006; Rothen and Meier, 2013); *Concurrent-Bandwidth: #* Concurrents/# Possible Concurrents	*Inducer-Bandwidth:* > 5 inducers for 36 letters and numbers; (e.g., Rothen et al., 2013a);*Concurrent-Bandwidth:* > 5 concurrents for 36 letters and numbers	We are in favor of using this low cut-off for the Inducer-Bandwidth, as it would include synesthetes who experience only vowels and/or numerals as having synesthetic colors. By definition, trainees with only four letter-color mappings would be excluded. The relationship between Inducer- and Concurrent-Bandwidth is unclear. We feel that Concurrent-Bandwidth should be addressed directly in future research in order to reach a consensus on the criterion. Using color charts can help differentiate, for example, whether two “reds” are actually the same color or only have the same category name
	Not “perceptually present”	Question the participant	The individual should not consider synesthetic concurrents to be “in the world” in the same way as the experience of the inducer (Rouw et al., 2013; Rouw and Ridderinkhof, 2014; Seth, 2014), although they do often feel as though they belong to the inducer. Synesthetic concurrent experiences are not confused with hallucinations, which are more complex, irregular, and unpredictable (Cytowic, 2002; Sagiv et al., 2011)	This does not exclude the possibility that the knowledge about the relationship of synesthetic concurrents to features of the real or external world may be *learned*, however, synesthetes do not consider their concurrent experiences to feel like *hallucinations* even though they do commonly describe them as real or an integral part of the experience. Importantly, visual hallucinations replace the visual field in which they “exist” rather than superimposing themselves or co-existing with the veridical percepts (Cytowic, 2002)
	Above criteria is not fake, made up, or due to “the expectancy effect”	The experimental goals should be kept as discrete as possible. Experimenters should be blind to the subject groups and true purpose of the experiment whenever possible. After testing, ask participants to openly report possible strategy use and their interpretation of the experiment	Prevented and verified *post-hoc*: Effects found in the other dimensions listed above should not be due to the participants consciously or unconsciously fulfilling the expectations of the experimenter. Exclude participants who admit to pretending to have subjective traits or trying to adjust their behavior in an unnatural way in accordance with what they believe the desired experimental goals are	We note that in practice, this is challenging to completely exclude, however, it is an important concern that we feel needs to be addressed
Major axis developmental grapheme-color synesthesia	Early age of onset	Question the participant (include family members and friends of the family if possible)	The synesthetic experiences have been or are reported to have been present since early childhood (age 3–10) or as far back as the individual can remember (Cytowic, 2002)	Developmental synesthetes can often report the first time they realized their experiences were different from the experiences of others
		Longitudinal research (Simner and Bain, 2013)	A developmental synesthete will show higher levels of consistency (compared to controls of the same demographic group) beginning in childhood and continuing into adulthood (Simner and Bain, 2013)	The percentage of fixed grapheme-color associations has been shown to increase with age (Simner et al., 2009; Simner and Bain, 2013)
	Not caused by pathology	Family history, medical records, questionnaires (e.g., Baron-Cohen and Harrison, 1997; Cytowic, 2002)	No history of disease, drug use, pathology or neuropathology	If synesthetic behavior and experiences persist following brain damage or drug use, then an individual may be considered to have acquired synesthesia, but not developmental synesthesia. It is not clear whether cases that may co-occur with psychological and cognitive phenomenon such as autism, schizophrenia, or other pathologies such as multiple sclerosis share the same underlying neuro-developmental trajectory as developmental synesthesia

*Methods, criteria, and references (when available) are listed for testing the necessary dimensions related to diagnosing synesthesia along the major axes: grapheme-color synesthesia (seven dimensions) and developmental grapheme-color synesthesia (nine dimensions). Some of the dimensions have multiple methodologies for testing the criteria. More research is needed to test whether different methodologies used within one dimension provide corresponding results. We propose at least one criterion per dimension should be met for an individual to be considered to have grapheme-color synesthesia*.

Diagnosing synesthesia *in a single individual* is based on a variety of characteristics. Concerning diagnosing “trained” forms of synesthesia, Rothen and Meier (2014) state: “Hence, to confirm the hypothesis, that synesthesia can be induced via training, would require the trained inducers to (i) *consistently* and (ii) *automatically* elicit (iii) the associated concurrent *experience* with perceptual qualities on a subjective phenomenological basis (iv) for the great majority of the inducers' occurrences (v) over an extended time period” (italics are the authors' own). We propose to extend their definition with a diagnostic criteria checklist.

We propose a guideline for diagnosing GCS based on dimensions along major axes for GCS (Table 1). Such a checklist can aid in the diagnosis of GCS (whether trained, acquired or developmental in nature). In this checklist, we present methods, criteria and references for the measurement of each dimension whenever possible. We propose that the presence of at least one result within each of the dimensions in Table 1 to be the necessary criteria for a diagnosis of GCS at the individual level. Several dimensions have multiple methodologies for testing the criteria. An important question that cannot yet be answered is whether the different methodologies give congruent patterns of results. For example, do individuals who report having stronger perceptual experiences also show larger perceptual crowding effects? Such issues are particularly relevant when testing trainees, where differences between measurements may emerge, such as the presence of a Stroop effect in the absence of conditioned response (Rothen and Meier, 2009).

The **Consistent** nature of the mappings involves the *Percentage* of mappings that do not change over a *Duration* of time (e.g., Baron-Cohen et al., 1987; Asher et al., 2006), or the *Level* of consistency as measured in color distance between repetitions of identical trials of a test (Eagleman et al., 2007; Rothen et al., 2013a). Although the consistency criterion is debated, the percentage and duration of consistency have traditionally been considered the “test of genuineness,” because of the inability of control participants to perform at the same level as synesthetes when all task demands are in their favor (e.g., Baron-Cohen et al., 1987; Asher et al., 2006). If the presence of synesthesia-like behavior or experience is temporary (e.g., drug use or hypnosis), the individual will be excluded over time by the consistency criterion.

The **Automatic** nature of synesthesia refers to the involuntary nature of the experience; it cannot willfully be “turned on or off.” (This involuntary nature does not necessarily imply that GCS is pre-attentional or unconsciously evoked, e.g., see Mattingley, 2009). Automaticity can be verified in behavior, with the commonly used “synesthetic Stroop task,” (Wollen and Ruggiero, 1983) or with a validated questionnaire (Rothen et al., 2013b), and in physiology as measured with pupil diameter (Paulsen and Laeng, 2006) or a color-sound conditioning paradigm (Meier and Rothen, 2009).

The **Conscious** nature of the experience of a percept in the absence of its external stimulation is perhaps the hallmark of synesthesia. Perception can be conscious or unconscious in neurobiological terms (e.g., Lamme and Roelfsema, 2000). Verifying the conscious perception of a synesthetic experience is typically the starting point of recruiting synesthetes to participate in a study. Synesthetic experiences are verified to be *conscious* by interview with the individual. Conscious experiences have been found related to consistent experiences (Simner et al., 2006). Trainees can be assessed with specifically designed questionnaires (Colizoli et al., 2012, 2014).

The **Perceptual** nature of the synesthetic experience can be objectively verified by using a variety of psychophysical visual tasks (e.g., Palmeri et al., 2002; Hubbard et al., 2005; Kim et al., 2006; Nikolić et al., 2007). Confirmatory findings where obtained with neuroimaging techniques (e.g., Nunn et al., 2002; Elias et al., 2003; Steven et al., 2006). Not all sub-types of synesthetes consistently show perceptual effects of synesthetic color when measured objectively (e.g., Hubbard et al., 2005; Rothen and Meier, 2009; Ward et al., 2010; Rich and Karstoft, 2013), and this is a point of discussion in diagnosing GCS (Rothen and Meier, 2014).

**Bandwidth** refers to the extent of the mappings between modalities and this variability leads to the idiosyncratic nature of GCS at the group level (Asher et al., 2006; Rothen and Meier, 2013). *Inducer-Bandwidth* refers to the proportion of synesthetic inducers compared to the number of possible inducers. *Concurrent-Bandwidth*, which has received relatively less attention in the field, refers to the proportion of synesthetic concurrents compared to the number of possible concurrents.

**Perceptual Presence**, which is the feeling that an experience is “real” in the same way that an event or object in the outside world is real, is characteristically *lacking* for synesthetes (Rouw et al., 2013; Rouw and Ridderinkhof, 2014; Seth, 2014). A synesthete typically does not get confused between sensations reflecting events in the outside world, and sensations caused by their synesthesia. Thus, for a synesthete, an inducer has perceptual presence, but the concurrent does not. This distinction is similar to the distinction drawn between synesthetic experiences and hallucinations (e.g., Cytowic, 2002; Sagiv et al., 2011); synesthetic experiences are notably simpler, more regular and more predictable than hallucinations.

Lastly, an important factor to consider that is often neglected is the “**Expectancy Effect**,” which is the reactivity on the participants part when they behave (consciously or unconsciously) in the way they believe they are expected to behave based on the characteristics of the study. This is an important and possibly confounding factor for any study where synesthesia is evoked (e.g., through learning, suggestion, or hypnosis). It may be the case that participants have guessed the purpose of training paradigms and then behave accordingly. Therefore, none of the effects related to “synesthesia” in the dimensions listed in Table 1 may be caused by the expectancy effect to be considered GCS.

Experiencing synesthesia since early childhood would be necessary for inclusion as **Developmental GCS**, but by definition would not be necessary for inclusion as trained or acquired GCS. Developmental GCS occurs in the absence of pathology, such as disease, drug use or mental disorders (Baron-Cohen and Harrison, 1997; Cytowic, 2002). Synesthetes differ from non-synesthetes in biological aspects such as functional and structural brain differences and the presence of certain genetic markers (Asher et al., 2009; Tomson et al., 2011). As previously mentioned, two main issues make the use of these markers unreliable for diagnosing synesthesia at the individual level. First, the group-level nature of such studies does not allow for inference at the individual level. Second, differences found at the neural and genetic levels may arise due to unknown causes in addition to causes that may be unique to synesthesia. Perceptual training paradigms may be suited to probe these underlying neural and genetic mechanisms.

Paradigms directed at training synesthesia can help to answer some open questions pertaining to the development of synesthesia, learning and memory, such as: Is there a critical period for the perceptual development of synesthesia? As far as we know, there are no developmental neuroimaging studies on synesthesia or training studies in children in potential critical periods of development. The brains of children show high levels of neuroplasticity, meaning high sensitivity to forming structural changes (Schlaug et al., 2009). It has been shown that specific synesthetic colors stem directly from childhood toys (Witthoft and Winawer, 2013), but this seems to be the exception, not the rule (Rich et al., 2005). Understanding the developmental pattern in synesthesia will help characterize the interaction between genes and environment in perceptual development.

In sum, we feel that trying to train “synesthesia” is a win-win situation. If GCS can be trained, we will have gained an understanding about who is more likely to become a synesthete and how. If GCS cannot be trained, we will still have gained an understanding about what the necessary and sufficient conditions must be in order to develop it. Our opinion is that trainees should not be considered to have acquired GCS *per se* unless the proposed criteria are met and should always be considered to be distinct from developmental synesthetes. We ask for and welcome the expansion and improvement of this diagnostic checklist. A commonly used model that can be both diagnostic and flexible is necessary for reaching a consensus in the field on the definition of (trained?) GCS.

## Conflict of interest statement

The authors declare that the research was conducted in the absence of any commercial or financial relationships that could be construed as a potential conflict of interest.
